# Artificial Intelligence-Driven Analysis of Antimicrobial-Resistant and Biofilm-Forming Pathogens on Biotic and Abiotic Surfaces

**DOI:** 10.3390/antibiotics13080788

**Published:** 2024-08-22

**Authors:** Akanksha Mishra, Nazia Tabassum, Ashish Aggarwal, Young-Mog Kim, Fazlurrahman Khan

**Affiliations:** 1 School of Bioengineering and Biosciences, Lovely Professional University, Phagwara 144001, Punjab, India; akanksha.42200033@lpu.in; 2 Marine Integrated Biomedical Technology Center, The National Key Research Institutes in Universities, Pukyong National University, Busan 48513, Republic of Korea; nazia99@pukyong.ac.kr (N.T.); ymkim@pknu.ac.kr (Y.-M.K.); 3 Research Center for Marine Integrated Bionics Technology, Pukyong National University, Busan 48513, Republic of Korea; 4 Department of Food Science and Technology, Pukyong National University, Busan 48513, Republic of Korea; 5 Institute of Fisheries Science, Pukyong National University, Busan 48513, Republic of Korea; 6 International Graduate Program of Fisheries Science, Pukyong National University, Busan 48513, Republic of Korea

**Keywords:** antimicrobial resistance, biofilm formation, artificial intelligence, machine learning, deep learning, image processing, microbial pathogens, biotic surfaces, abiotic surfaces

## Abstract

The growing threat of antimicrobial-resistant (AMR) pathogens to human health worldwide emphasizes the need for more effective infection control strategies. Bacterial and fungal biofilms pose a major challenge in treating AMR pathogen infections. Biofilms are formed by pathogenic microbes encased in extracellular polymeric substances to confer protection from antimicrobials and the host immune system. Biofilms also promote the growth of antibiotic-resistant mutants and latent persister cells and thus complicate therapeutic approaches. Biofilms are ubiquitous and cause serious health risks due to their ability to colonize various surfaces, including human tissues, medical devices, and food-processing equipment. Detection and characterization of biofilms are crucial for prompt intervention and infection control. To this end, traditional approaches are often effective, yet they fail to identify the microbial species inside biofilms. Recent advances in artificial intelligence (AI) have provided new avenues to improve biofilm identification. Machine-learning algorithms and image-processing techniques have shown promise for the accurate and efficient detection of biofilm-forming microorganisms on biotic and abiotic surfaces. These advancements have the potential to transform biofilm research and clinical practice by allowing faster diagnosis and more tailored therapy. This comprehensive review focuses on the application of AI techniques for the identification of biofilm-forming pathogens in various industries, including healthcare, food safety, and agriculture. The review discusses the existing approaches, challenges, and potential applications of AI in biofilm research, with a particular focus on the role of AI in improving diagnostic capacities and guiding preventative actions. The synthesis of the current knowledge and future directions, as described in this review, will guide future research and development efforts in combating biofilm-associated infections.

## 1. Introduction

The increase in mortality rates due to microbial pathogen infections has become a global health concern [1,2,3]. The development of antimicrobial resistance (AMR) mechanisms causes failures in treating such infections [4]. Microbial pathogens exhibit various mechanisms of AMR [5,6]. Among these, biofilm formation is a major resistance mechanism primarily used by bacterial and fungal pathogens [7,8,9].

Biofilms are formed by a collection of microbial cells enveloped by extracellular polymeric substances (EPS) produced by these cells [10,11]. EPS is composed of polymeric molecules such as nucleic acids, polysaccharides, proteins, and lipids [12,13]. EPS confers several benefits to microbial cells, such as enhanced cohesion and adhesion onto biotic or abiotic surfaces, a spatial structure, and facilitated interactions between microbes [14,15]. The biofilm structure further hinders the penetration of antibiotics/antifungals or disinfectants into the microbial cells and thereby allows them to survive without any effect on the external environment [16,17]. In addition, the biofilm environment also facilitates the generation of mutant and dormant (persister) cells, which exhibit a high rate of resistance and tolerance against antimicrobial agents [18,19,20].

**Figure 1:**
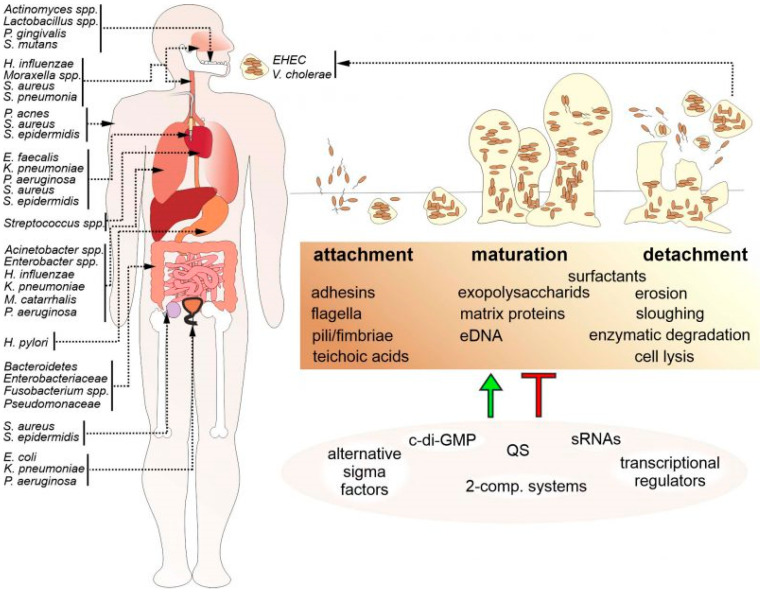
The biofilms created by bacterial pathogens are present on various human body tissues, medical equipment, such as catheters or prostheses, and in the environment. It acts as a reservoir for potential infections that may occur in the future. The common bacterial species that are associated with diseases caused by biofilms are depicted in a schematic figure on the left. The arrows in the diagram indicate where these bacteria are located throughout the body. When it comes to the development of biofilm, attachment, maturation, and separation are all essential components (bottom right). Multiple components contribute to this multistep process, including bacterial surface chemicals, secreted matrix effectors, ambient components, and stressors. Therefore, the regulation of bacterial biofilm (located on the lower right) necessitates the utilization of intricate positive and negative regulatory mechanisms. Second messengers such as Bis-(3′-5′)-cyclic dimeric guanosine monophosphate (c-di-GMP) are examples of these mechanisms. Quorum sensing (QS), regulatory sRNAs, alternative sigma factors, two-component systems, and other mechanisms are also included. Reprinted from [26], Copyright © 2021 by the authors and Shared Science Publishers OG. Enterohemorrhagic *Escherichia coli* (EHEC), extracellular DNA (eDNA), and small RNAs (sRNAs).

Because microbes are ubiquitous in various environments, biofilm formation on both biotic and abiotic surfaces is highly likely [21,22,23]. Biofilms can be formed in various tissues and organs in the human body, including the oral cavity, middle ear, upper respiratory tract, lungs, stomach, female reproductive organs, urogenital system, cardiovascular system, colon, bone, contact lenses (CLs), and soft tissue wounds [24,25,26] (Figure 1). The proposed technique classifies various species of bacterial biofilms from in vitro cultures and clinically obtains in vivo images from human subjects with otitis-media-related bacterial biofilms from optical coherence tomography images by applying supervised machine learning (ML)-based frameworks (Support Vector Machine (SVM), Random Forest (RF), and XGBoost) [27]. This study explores novel ideas and modern strategies for controlling and avoiding biofilm formation on CL. In addition to the difficulties presented by biofilms on CL, this study investigates the developments in biofilm detection methods and their practical applications [28].

The formation of biofilms by microbial pathogens on fresh foods and vegetables is also detrimental to human health [29]. Biomedical devices such as pacemakers, shunts, prostheses, catheters, CLs, surgical instruments, urinary catheters, and endoscopes are highly prone to biofilm formation by microbial pathogens [30,31]. Furthermore, the formation of biofilms by microbial pathogens on various food surfaces has also been reported, which represents a huge burden on the food industry [32]. The food industry employs a variety of materials, including polyethylene, wood, stainless steel, glass, rubber, and polypropylene, for different purposes, such as the storage, transport, and packaging of food items [33]. These surfaces also act as surfaces for the establishment of microbial biofilms. The formation of biofilms by microbial pathogens on the surfaces of food, food materials, and processing equipment often results in food spoilage, infections, alteration of food ingredients, and corrosion of metal surfaces [34].

Beyond increasing the awareness of the necessity to control biofilm-forming pathogenic microbial infection, precise detection and identification of these biofilms and associated pathogens on biotic and abiotic surfaces is also required to establish treatment strategies [35]. Delayed diagnosis of biofilm infections by microbial pathogens necessitates the administration of high doses of antibiotics in large quantities, which, in turn, fosters the growth of antibiotic-resistant bacterial strains [36]. Consequently, there is an immediate need for systems that can promptly identify and manage biofilm infections [37]. A rapid detection method in this regard relies on crystal violet (CV) staining to examine the presence of biofilms [38]. Similarly, fluorescence microscopy is also used to detect microbial biofilms [39]. Electron microscopy has enabled advanced imaging of biofilms on various surfaces [40]. Within water flow systems, a sensitive electrical resistance spectroscopy technique is also used to track the development of biofilms across metallic substrates in real time and non-invasively. Using this technique, *Pseudomonas fluorescens* single-strain biofilms were developed in a lab-scale test setting under laminar flow and constant temperature conditions [41].

However, despite the progress made in biofilm detection, precise detection with high-resolution images that can clearly distinguish different species of microbial colonies in the biofilm has failed [10]. Artificial intelligence (AI) techniques are being increasingly applied in different areas, such as medicine, the food industry, dental devices, and engineering. Through image analysis, tailored treatment planning, real-time monitoring, and therapeutic discovery, AI has the potential to improve the efficacy of antimicrobial photo–sonodynamic therapy in the treatment of inflammation in oral healthcare systems [42,43]. Indeed, significant research is being conducted to control oral biofilm formation by microbial pathogens, which can lead to several systemic illnesses. To this end, an AI-based, computer-assisted detection system based on a deep convolutional neural network (CNN) architecture was demonstrated to accurately detect dental biofilms [44]. Reports on the implications of AI tools and techniques for advancing the detection of microbial pathogens have also been published [45]. This highlights the presence of biofilms in food-producing chains, as well as the fundamental intracellular processes behind illnesses linked to biofilms. An improved understanding of microbial biofilms can indeed be achieved using ML techniques [46]. Therefore, utilizing biotechnological technologies to combat the creation of biofilms and eradicate biofilms in the food sector poses a severe threat to human health could be possible [47]. Because metal-organic frameworks have an ordered structure, adjustable composition, easy synthesis, adjustable porosity, simple functionalization, high drug loading, and good biocompatibility, they have been used extensively in biofilm therapy to address issues with different biofilm treatment methods, such as significant side effects and limited efficacy [48]. Several studies have employed AI methods to investigate the impact of various environmental factors on biofilm formation by microbial pathogens [49]. This review aims to provide a comprehensive overview of AI-based detection of biofilm-forming microbial pathogens on both biotic and abiotic surfaces [50]. In addition, the review proposes several future perspectives that form the basis for the application and role of AI in the biomedical, clinical, food, and agricultural industries [51].

## 2. Clinical Significance of Biofilm-Forming Microbial Infection

Biofilm-forming microbial infections represent substantial clinical concerns since they are generally resistant to antimicrobial therapies and can cause serious illnesses [52]. These infections are particularly challenging to manage because of their chronic nature and growing drug resistance [4]. Biofilms are implicated in over 60% of human infections, underscoring their role in public health [53]. In the USA alone, healthcare costs exceed USD 7.2 billion annually due to these infections, which contribute to substantial rates of morbidity and mortality [54]. For instance, fungal infections were responsible for more than 13,000 deaths in the USA between 2020 and 2021, exacerbated by conditions such as COVID-19 [55]. Similarly, bacterial antimicrobial resistance resulted in approximately 4.95 million deaths globally, highlighting a critical public health issue [56]. The World Health Organization (WHO) has prioritized identifying new antimicrobial agents to combat these pathogens, given the increasing risks associated with microbial resistance [57].

**Figure 2:**
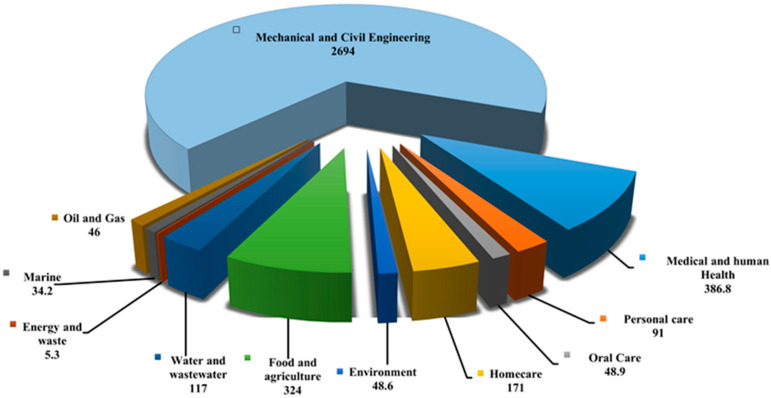
Global annual economic burden of various market sectors with biofilm-associated technologies (all values are in billions of dollars). The information obtained from [68].

**Figure 3:**
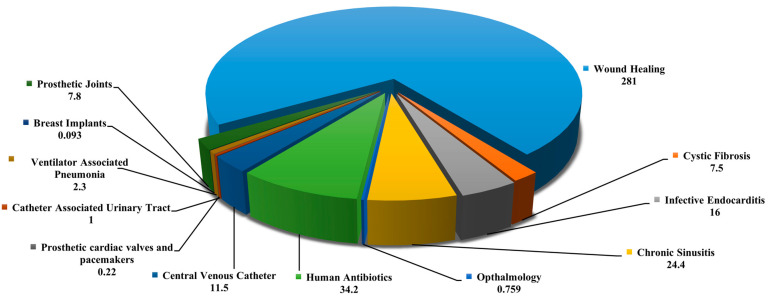
Global annual economic burden of various biofilm-associated infections on the health sector (all values are in billions of dollars). The information obtained from [68].

Pathogenic microbial biofilms represent a significant threat to the healthcare industry due to their resilience against traditional antimicrobial treatments [58]. Microbes in biofilms, existing in sessile forms, exhibit phenotypic differences that make them up to 1000 times more resistant than their free-floating counterparts [59]. This resistance is linked to poor metabolic activity, limited antimicrobial penetration, persister cells, antibiotic-modifying enzymes, efflux pumps, extracellular DNA, and horizontal gene transfer mechanisms. This resistance is attributed to factors such as low metabolic activity, limited antimicrobial penetration, persister cells, antibiotic-modifying enzymes, efflux pumps, extracellular DNA (eDNA), and horizontal gene transfer mechanisms. Difficulties in antimicrobial diffusion through biofilm matrices, microbial inactivation rates, and environmental protection mechanisms further contribute to this resistance [60]. Common biofilm-forming pathogens include *Pseudomonas aeruginosa*, *Candida albicans*, *Acinetobacter baumannii*, and *Staphylococcus aureus*, among others, known for their ability to colonize medical devices and tissues, thereby increasing infection risks [61,62]. Biofilms present a substantial economic burden globally, amounting to approximately USD 5 trillion annually across various industries [63,64]. In clinical settings, managing wounds associated with biofilm-forming pathogens like *S. aureus*, *P. aeruginosa*, and *C. albicans* incurs significant costs. For example, treating chronic wounds linked to biofilm infections costs approximately USD 96.8 billion annually in the USA and GBP 8.3 billion in the UK [65]. Chronic wounds are predominantly attributed to biofilm-associated infections, accounting for a substantial portion of global wound care expenditures [66]. In conditions like cystic fibrosis, *P. aeruginosa* biofilms in the lungs contribute to persistent infections resistant to conventional therapies, significantly impacting healthcare costs [67,68]. Recently, Cámara et al. [68] emphasized the considerable economic impact of microbial biofilms across various sectors and outlined the major scientific and technological challenges that need to be tackled to fully exploit opportunities for translational research in this field (Figure 2 and Figure 3).

## 3. Limitations of Detection and Analysis of Biofilms Using Biochemical and Microscopic Techniques

BiofilmQ, ImageJ, BioFilm Analyzer, and Imaris are examples of microscopy image analysis tools that have been successfully utilized to evaluate the geometric dimensions of cell features (e.g., using images to determine the length of a rod-shaped bacterial cell) and for microscopy image feature extraction [69,70,71]. These strategies work well when the features in the microscope images are homogeneous and do not overlap. However, they fail with crowded features such as overlapping bacterial cells. Biofilms can also show heterogeneities in terms of bacterial cell size and shape, cell clusters, holes, and microbial detritus in microscopy pictures [72].

The ultrastructure of a biofilm can be characterized using microscopy, with the highest magnification being offered by scanning electron microscopy (SEM) [73]. It is also not necessary to use traditional SEM when high-resolution images depicting the true biofilm ultrastructure are required; in this case, a novel OsO4-RR-TA-IL procedure is advised [74]. Using this technology, the biofilm extracellular matrix is made conductive to the electron beam, enabling the viewing of bacterial cells as if they were implanted in the matrix and the assessment of their subsurface structures [75]. By avoiding drying and dehydration, the details of the EPS structure are also revealed. The three-dimensional (3D) ultrastructure biofilm matrix can be quantitatively evaluated using a combination of cutting-edge SEM methods and 3D image analysis tools [76]. The development of effective anti-biofouling solutions requires the use of monitoring systems because irradiation biofouling poses issues for bioreactors and other sectors. These surveillance methods are referred to as “biofilm detection techniques” [77].

Traditional methods of identifying biological destructors involve probing surfaces to prepare them for further laboratory studies. This method is costly, time-consuming, and requires the involvement of specialists [78]. Another method relies on sensor-based biofouling measurements. These sensors are based on various physical concepts, including luminescence effects and light intensity measurements to internal light reflection effects [79]. The suggested technique is based on the variation in the spectral band absorption and reflection of light by biological materials. The spectra of various biological components differ from one another. This previously allowed us to determine the type of biological object based on its spectral properties [80]. This method was applied to the processing of Earth’s surface images from space. The most important properties of biological items that enable the detection of differences between them are obtained through the analysis of photographs captured in the visible and near-infrared spectral bands [81]. In a previous study, electrical impedance spectroscopy (EIS) was used to track the growth of *Staphylococcus epidermidis* RP62A on the surface of gold-plated polyimide. *S. epidermidis* is a component of the normal human skin flora [82]. This bacterial species is an opportunistic pathogen that forms biofilms under specific environmental conditions. It has been associated with a significant number of hospital-acquired infections, primarily urinary catheter-related infections [83]. Unlike conventional impedance microbiology measurements, EIS measures the impedance response over a broad frequency range. Additionally, the data can be modeled using an equivalent circuit and interpreted in terms of the electrochemical reactions occurring within the system. The techniques are considered expensive due to high sensor costs [84].

Numerous techniques are available to quantify *Candida* biofilm formation. Multiple isolates can be identified quickly and reliably using techniques performed in 96-well polystyrene plates [85]. The most popular methods involve measuring biomass production using CV staining and measuring the metabolic activity of buried viable biofilm cells based on the reduction of the tetrazolium salt (sodium 3′-[1-(phenylaminocarbonyl)-3,4-tetrazolium]-bis (4-methoxy6-nitro) benzene sulfonic acid hydrate) (XTT) to formazan dye [86]. Previous studies involved small numbers of isolates and included only *C. albicans* isolates, yet all have indicated poor agreement between the two approaches [87]. The microtiter plate dye staining (MPDS) method uses culture plates and monitors the process of biofilm production and the activity of the generated biofilm. However, in situ identification of biofilms within a bioreactor is not possible [88].

Surface scattering and electron absorption form the basis of SEM, which enables high-depth imaging. Therefore, the surface of the biofilm appears 3D, and the distribution and dispersion of EPS on the biofilm can be observed [89]. The ability of SEM to magnify materials to the level of a single molecule enables the observation of the adhesion characteristics of individual bacteria. Hence, SEM is a useful tool to study materials that can repel biofouling films [90]. Extremely high levels of vacuum and drying of the sample are required to observe these characteristics. SEM is an ex situ and destructive technique that requires collecting and processing a biofilm sample. The sample may receive damage during this process [91].

Colony-forming units (CFU) are the most commonly used method to determine the viability of biofilm cells [92]. The core idea is to establish colonies of individual cells separated on an agar plate to distinguish between living and dead cells [93]. The inability of viable but non-culturable cells to grow on regular agar prevents CFU detection. As the CFU approach usually does not require sophisticated or specialized equipment, any microbiological laboratory can easily use it [94]. However, the CFU method also has drawbacks: it is very time-consuming, laborious, and prone to mistakes throughout the scraping and counting processes [95]. Fourier-transform infrared (FTIR) spectroscopy is often used as an alternative owing to its sensitivity and stability. The method uses infrared radiation, which is a non-destructive and non-invasive form of radiation. The covalent bonds between the biofilm parts vibrate as a result of IR [96]. Every sample has a certain range of variation spectrum because each component of the biofilm vibrates at distinct frequencies. FTIR spectroscopy was previously used to assess the progression of biofilms in patients with long-term wounds [97]. To detect various proteins and bacteria and make inferences regarding their time-varying quantities, the exudate from a chronic lesion was collected and placed under a spectrometer [98]. Despite its advantages, FTIR spectroscopy can only detect changes in the surface layer of biofilms and cannot determine their thickness or 3D structures [99].

## 4. Application of Artificial Intelligence for the Analysis of Biofilm of Microbial Pathogens on the Biotic and Abiotic Surfaces

**Figure 4:**
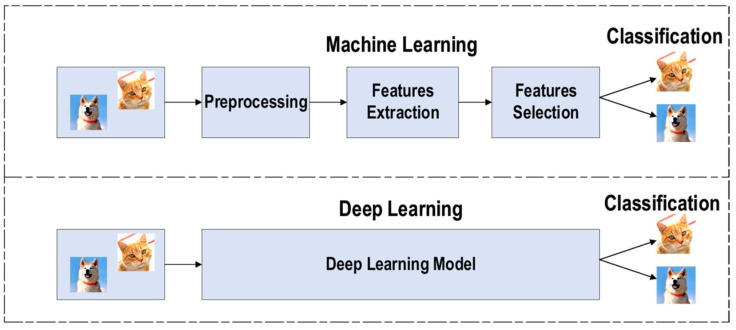
Basic block diagram of machine learning and deep learning. Reprinted from [105]. Copyright © 2021 by the authors and Licensee, J Big Data.

**Figure 5:**
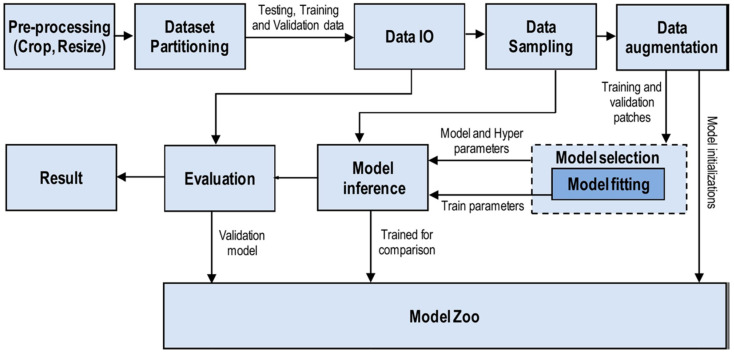
Internal workflow diagram of deep learning. Reprinted from the [105] Copyright © 2021 by the authors and Licensee, J Big Data.

**Figure 6:**
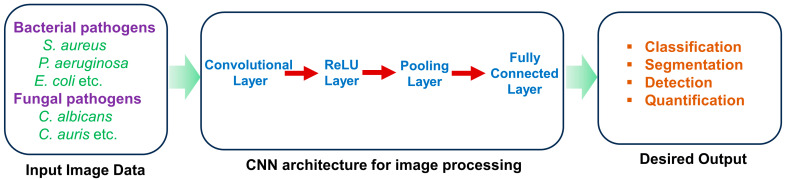
Deep learning architecture represents the detection of microorganisms from microscopic images.

AI techniques have attracted significant global attention in recent years. Coined by John McCarthy in 1956 as “the field of science and technology of developing intelligent devices, especially intelligent computer programs” [100], AI has shaped into a burgeoning research field. John Searle further delineated AI as either ‘strong’ or ‘weak’ (narrow), contingent on whether machines can achieve consciousness [101]. Currently, AI applications focus primarily on solving specific problems and tasks in science. The latest phase of the industrial revolution is also characterized by the integration of AI and big data across numerous businesses. Key AI methodologies, such as ML and deep learning (DL), play crucial roles in achieving diverse objectives. They are extensively used in robotics, natural language processing, image analysis, signal processing, and speech recognition (Figure 4, Figure 5 and Figure 6). AI algorithms have revolutionized medical imaging, enabling the automatic segmentation of radiological images such as X-rays, CT scans, MRI scans, PET scans, and ultrasound images [102]. AI-driven segmentation significantly enhanced the accuracy and efficiency of tumor and cancer diagnoses in human organs [103,104].

Applications of AI in science include predicting the likelihood of composite resin crown debonding, assisting in orthodontic treatment planning, electroencephalographic analysis, electrocardiography-based disease detection, genomic research, protein sequencing, and also detection of the structural attributes of bacterial biofilms from microscopy images [106,107,108]. In this investigation, the surface roughness of cast iron (7.10 ± 0.78 nm) is higher than that of PVC (5.60 ± 0.14 nm), and its positive charge creates an environment that is conducive to the production of biofilms. A CNN was constructed to predict the growth and physical characteristics of fouling layers [109]. Biofilms can be formed by various species of gram-positive and gram-negative bacteria [110]. The accurate detection of the type of bacteria within biofilms is crucial for numerous clinical applications because the type of bacteria (gram-positive or gram-negative) and surface type (biotic or abiotic) significantly influence the characteristics of biofilms. Researchers have developed diverse AI algorithms to address various aspects of biofilms, including the prediction of biofilm formation, identification of genes and gene structures, and evaluation of the effectiveness of new therapeutic approaches. AI algorithms have also been used to analyze the surface properties of *Desulfovibrio alaskensis* G20 bacterial biofilms on mild steel using SEM [72]. Techniques such as deep convolutional networks (DCNNs) and mask region convolutional neural networks (M-RCNNs) have been adapted for SEM image segmentation, with M-RCNNs and DCNNs proving to be 227 and 70 times faster, respectively, than traditional methods. Validation studies demonstrated that M-RCNN is 1.06 times faster than classical ImageJ-based analysis. Using a dataset of 66 SEM images, the researchers identified key biofilm characteristics. The encoder component of the DCNN captured multiscale contextual information from input images, while M-RCNN was built around a pre-trained backbone neural network, such as ResNet101 [111].

In other studies, advanced DL models, such as Barlow Twins and MoCoV2, achieved superior accuracy (8% and 6% improvement) compared to the supervised ResNet-50 model on a small dataset of seven grayscale images [112]. Another innovative CNN model-based biofilm scanner, YOLACT, has been developed to detect *Desulfovibrio alaskensis* G20 bacterial biofilms on steel surfaces [113]. Results indicated that these biofilm scanners provide response times 2.1 and 6.8 times faster than the M-RCNN and DLv3+ AI models, respectively. Machine learning based AI models has also been considered in cetacean image identification [114]. In dentistry, the UNet neural network is used to automatically detect biofilms in tooth images. The UNet model achieved a high accuracy of 91.8%. Additionally, the Fiji software (https://fiji.sc, accessed on 18 August 2024) equipped with an ML plugin has been utilized to directly analyze bacteria using SEM images of structured and non-structured surfaces [115]. This approach has enabled the direct analysis of SEM images to assess bacterial morphology and quantify bacterial adhesion on various dental material surfaces, offering a simplified and cost-effective AI-enabled method.

A CNN trained for automatic image recognition has yielded significant findings in rhinology, particularly in the analysis of bacterial cells within the nasal mucosa [116]. The model achieved 98% accuracy in analyzing the texture of nasal mucosa using microscopic images. Artificial neural networks (ANNs) demonstrate the potential to analyze, validate, and predict the biosynthesis of *Chitosan nanoparticles* (CNPs) using extracts from *Olea europaea* leaves as well [117]. These CNPs have shown capabilities to suppress biofilm formation by pathogens, such as *P. aeruginosa*, *S. aureus*, and *C. albicans*.

AI-driven DL models are also proficient in analyzing microscopic images of various microorganisms, including viruses, bacteria, and fungi [118]. A comprehensive study compared seven different architectures of deep CNNs to classify 1079 microscopic images from 89 fungal genera (e.g., *Candida auris* and *Aspergillus terreus*). Among these architectures, DenseNet achieved the highest accuracy at 79%. Moreover, deep neural networks have been employed to detect the thickness of biofilms in desalination plants to aid prevention and control measures [119]. Using a dataset of 300 optical coherence images, the algorithm achieved a remarkably low mean square error of 0.008 µm^2^ in determining the biofilm thickness [120]. In another healthcare application, ANNs were used to categorize glomeruli classes in kidney biopsy images from 26 images of 19 donors, achieving an accuracy of 99%. This algorithm holds promise for clinical applications aimed at effectively categorizing kidney biopsy images.

**Figure 7:**
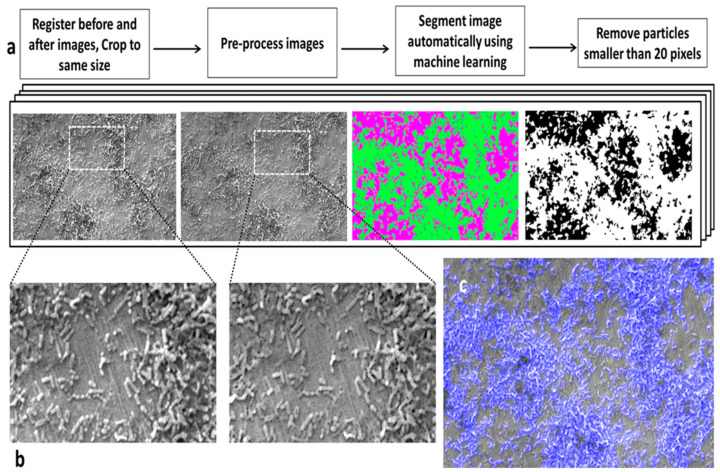
(**a**) A method was devised to segment biofilm from surfaces in SEM images. (**b**) Close-up images illustrate the surface before and after pre-processing, where outliers were eliminated to smooth scratches on the surface, facilitating improved segmentation. (**c**) An overlay demonstrates the segmented area (highlighted in blue) overlaid onto the SEM image, showcasing precise biofilm detection. Reprinted from [121]. Copyright © 2016 by the authors and Licensee, Scientific Reports.

Addressing the build-up of biofilms on biomaterials is a critical concern, prompting extensive efforts to develop effective removal strategies. AI algorithms have been used to assess the efficacy of biofilm-removal techniques [121]. For instance, efforts to disrupt *Streptococcus mutans* biofilms on titanium and polished titanium substrates utilized ML algorithms with the Fiji software to evaluate their performance using SEM images of the affected surfaces. Interestingly, the results indicated a lower accuracy for biofilm removal on titanium surfaces than on polished titanium. Moreover, combating antibiotic resistance is increasingly supported by the application of anti-biofilm agents [121]. However, the identification of the effective compounds remains challenging. Five ML algorithms—SVM, RF, Multilayer Perceptron (MLP), KStar, and M5Rules—were assessed to determine the efficiency of anti-biofilm chemicals based on IC_50_ levels [122]. Evaluation of three feature selection approaches—Support Vector Regressor (SVR), Decision Tree (DT), and Perceptron—revealed that SVM with SVR feature selection yielded the highest performance, achieving a Pearson’s correlation coefficient of 0.84. Additionally, the M-RCCN model demonstrated its utility in identifying bacterial communities using soil samples from various locations, including Greenland, Sweden, and Kenya [123]. Furthermore, the M-RCCN technique was used to study cell and colony morphologies, including metrics such as cell size, shape, and aggregation levels, using distance-to-nearest-neighbor computations. Deep neural networks have also been employed to identify patterns of bacterial colonization [124]. Utilizing microscopy images of the surfaces of sulfide minerals, three bioleaching bacterial species—*Acidithiobacillus caldus*, *Leptospirillum ferriphilum*, and *Sulfobacillus thermosulfidooxidans*—were detected with an accuracy of 90%, significantly surpassing the accuracy of conventional methods [125]. ML algorithms have demonstrated promising results in identifying biofilms on surfaces within agro-food-processing facilities as well (Figure 7). A previous study evaluated the feasibility of identifying *E. coli* and *Salmonella typhimurium* based on fluorescence hyperspectral imaging results. Comparative analysis of three ML algorithms—linear discriminant, k-Nearest Neighbor (k-NN), and partial least squares (PLS) discriminant analyses—indicated that k-NN analysis could rapidly detect biofilms with 90% accuracy.

Furthermore, ML techniques have demonstrated success in detecting the biofilm-modulating effects of essential oil components on the surface of polystyrene plates in 96-well microtiter plates [126]. Researchers evaluated six machine-learning algorithms—RF, Logistic Regression (LR), SVM, Gradient Boosting (GB), Decision Trees (DT), and k-NN—to detect *P. aeruginosa* strains. GB exhibited the highest accuracy (~100%) among all models. Similarly, in another study focusing on identifying *S. aureus* strains, GB also demonstrated the highest accuracy w ~ 100%) among the six ML algorithms assessed [127].

ML algorithms have also shown efficacy in analyzing the properties of complex surfaces of biological materials [128]. k-NN, Gaussian naïve Bayes, LR, and RF classifiers were used to classify the three different variants of *Bacillus subtilis* on MSgg agar. The k-NN outperformed all classifiers. ML algorithms can also be applied for detecting the inhibition of biofilms [129]. RF-based descriptors, fingerprints, and hybrid classifiers were used to detect the biofilm inhibition properties of the molecules using their chemical and structural features. RF-based descriptor classifier yielded the best results, with a classification accuracy of 93%. Researchers have also reported an ML-aided cocktail test for biofilm identification using multiplexing fingerprint physicochemical features [129]. *S. aureus, A. baumannii, P. aeruginosa, Stenotrophomonas maltophilia*, and *E. coli* biofilms were identified using a principal component analysis-based ML algorithm (Table 1). Transmission electron microscopy images of the biofilms were used to train and test the algorithm, which achieved a high level of accuracy (95%). Additionally, supervised ML algorithms were effective in predicting the combined effects of antimicrobial drugs and peptides [130]. The study utilized data related to the antimicrobial peptide features, which were collected from the database of antimicrobial activity and structure of peptides (BBAASP) [131]. In this database, microbial characteristics of 17 different species were identified, with the highest prevalence of *P. aeruginosa* (32.2%). Then, the performance of the 17 ML classifiers was compared, and the results showed that the hyperparameter-optimized Light Gradient Boosted Machine Classifier (oLGBMC) had the highest test accuracy of 76.92% for predicting the synergistic impact. Furthermore, the CNN-based segmentation DL algorithm efficiently predicted the 3D images of biofilm communities using confocal microscopy [132].

## 5. Application of Artificial Intelligence for Detection of the Effects of Various Environmental Factors on Biofilm Formation by Microbial Pathogens

**Figure 8:**
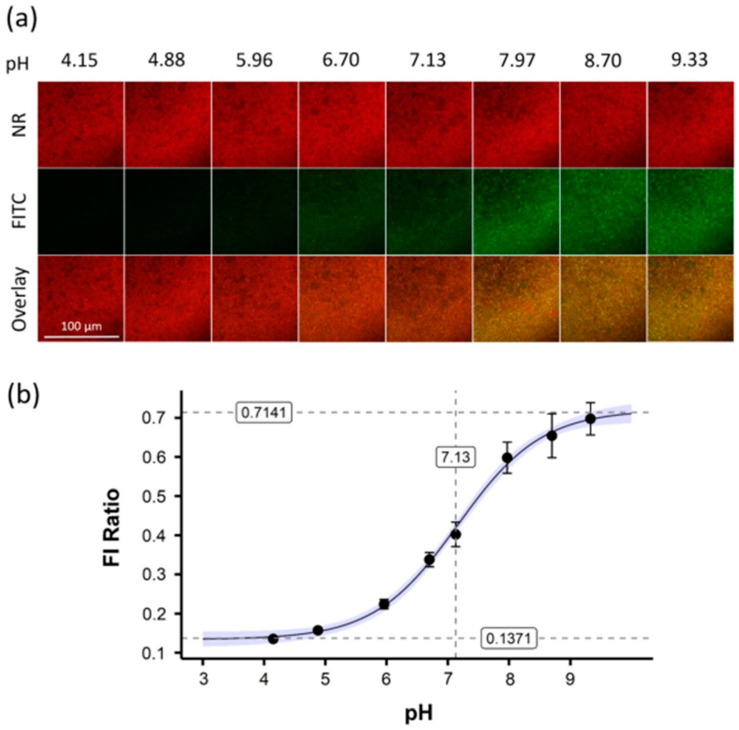
Confocal laser scanning microscopy allowed nanosensor fluorescence imaging in reference buffers. (**a**) FITC (green) and NR (red) nanosensor fluorescence were observed in 8 reference buffers. Z-stack imaging captured biofilm depth at each pH. Representative 2D pictures with overlays improve clarity. A 100 µm scale bar is used for all pictures. (**b**) FITC/NR fluorescence intensity (FI) was plotted versus pH. Error bars represent standard deviation, while blue-shaded regions represent fitted curve confidence intervals. Reprinted from [146]. Copyright © 2022 by the authors and Licensee, Scientific Reports.

The food, environment, and biomedical industries are sectors where biofilms can have a detrimental impact. The growth and development conditions of bacteria, including pH, temperature, and nutrients, may significantly influence biofilm formation (Figure 8) [133]. The development of novel microbiological techniques may help us understand how chemical and environmental variables affect biofilm formation [134]. Biofilm formation can be quantified using experimental approaches, and the impact of an extensive range of distinct environmental conditions on biofilm formation can be examined using mathematical models [135]. Researchers have studied the effects of pH, ethanol, and NaCl concentrations on biofilm growth [136]. The growth of *S. aureus* has been investigated because it is an opportunistic pathogen with a biofilm-forming ability that causes significant problems in healthcare. Regression analysis is based on the assumption that all essential data are available at the time of initial evaluation, which presents a challenge for studies employing this method. Some data may not be completely available because of laboratory limitations, which will exclude missing data in the linear regression. Consequently, the absence of this significantly reduces the accuracy of regression algorithms. For this reason, biofilm growth was evaluated using an ANN with feed-forward and backpropagation [136]. This model can represent complex nonlinear realities (for which there are insufficient physical and mechanical models) [137]. Growth was investigated at four pH levels, four NaCl concentrations, and four ethanol concentrations. ANN is a widely used DL model because of its ability to model data under complex environmental conditions [135,136,138,139]. The results indicated that all three factors affected biofilm formation. The influence of environmental factors (pH, water activity, and ethanol) was evaluated using a cardinal parameter model for *S. aureus* [135]. The results indicated that the model was efficient in identifying the effects of these factors on biofilm formation.

ANN is also effective in evaluating the effects of temperature on the microbial spoilage of meat [140]. In an analysis involving five temperature levels varying from 0 to 25 °C, feed-forward ANN and PLS models were trained and tested on images collected by FTIR to classify meat products. The training classification accuracy was observed using the ANN model, which shows the correlation between temperature and food quality. In another study, an ANN was used to identify the effects of temperature, pH, and NaCl on microbial activity [141]. Environmental factors also play important roles in the growth of antibiotic-resistant Salmonella [141]. Regression neural networks (RNNs) and Monte Carlo simulation models were compared to evaluate the effects of serotype and temperature. The RNN model outperformed the Monte Carlo simulation with an accuracy of 92%. The independent effects of temperature and serotype factors were also identified. The results showed that temperature and serotype influence the growth of *Salmonella* at rates of 48.3% and 16.8%, respectively.

Other AI models, such as the multilayer perceptron neural network (MLP-ANN) and radial basis function network (RBFN), have also shown good efficiency in detecting environmental factors affecting *Fusarium culmorum*-contaminated seeds [139]. The effects of the water activity, temperature, time, and inoculum size were investigated. The results showed that depending on the training algorithm, the single-layer perceptron model with a small number of hidden nodes outperformed the double-layer perceptrons. The RBFN was able to achieve lower errors and better generalization than MLP-ANN, although it required a significant number of hidden nodes to achieve these results. AI models have also been used to investigate bacterial growth in wastewater [142]. In this study, an ANN model was examined to determine whether it could forecast the growth of a bacterial strain known as *Klebsiella* sp. during the treatment of wastewater polluted with diclofenac sodium. In order to conduct batch examinations, input parameters, including temperature, pH, time, agitation, and concentration of diclofenac sodium, were randomly combined. Compared to the multiple linear regression model, the ANN model demonstrated a higher level of efficiency. Additionally, in another study, ANN demonstrated the capability of determining the diameter of the inhibitory zone formed by walnut extract on 12 different types of bacteria [143].

Hiura et al. [144] conducted a complex meta-analysis to determine the effects of environmental conditions on bacterial biofilm formation. Population growth and inactivation data of *Listeria monocytogenes* were collected under 1007 distinct environmental circumstances. These conditions included five dietary categories: beef, culture medium, pork, seafood, and vegetables. Another AI model, an extreme GB tree, was used to forecast bacterial population behavior based on eight explanatory variables: time, temperature, pH, water activity, initial cell counts, ‘if the viable count is the initial cell number’, and two food categories. The proposed model efficiently classified the effects of all conditions on biofilms. The DL model accurately detected the impact of environmental conditions on the growth of different strains of *Bacillus* species [138]. A total of 317 scenarios were used to evaluate the effectiveness of the model. These conditions included nine pH levels, six water activity levels, six acetic acid concentrations, and five lactic acid concentrations. The results were compared with those of an LR model. The DL model made more accurate predictions of independent data with lower probabilistic variability values than the LR and neural network models. To control bacterial development in a manner that is both secure and adaptable, DL methods can be utilized for growth boundary modeling. The growth kinetics of acetic acid-producing bacteria isolated from fruit waste were analyzed using five different ML models: multivariate linear regression, PLS regression, kernel ridge regression, support vector regression, and GB regression [145]. The microbial growth kinetic analysis was conducted at different glucose concentrations (1–5%). Based on a comparative analysis, the gradient-boosting regression model was found to outperform the other models in terms of growth kinetics prediction.

## 6. Conclusions and Future Perspectives

Biofilm-associated infections provide important therapeutic problems owing to their antibiotic resistance and chronic nature. These infections not only significantly increase healthcare expenses and morbidity rates globally, but they also raise the risk of infection by colonization of medical equipment and tissues. This demonstrates their significant influence on public health and the healthcare economy. Biofilms illustrate a variety of resistance mechanisms, including low metabolic activity, limited antimicrobial penetration, persister cells, antibiotic-modifying enzymes, extracellular DNA, and horizontal gene transfer, making them very resistant to traditional therapy. The economic impact is significant, with yearly healthcare expenses estimated in the billions, underscoring the critical need for effective management measures and novel therapeutic options to enhance patient outcomes. The current biochemical and microscopic methods for identifying and studying biofilms have limitations. For example, SEM provides deep structural insights, although it requires advanced sample preparation and is often conducted ex situ. Traditional approaches, such as colony-forming unit counts, are labor-intensive and can overlook live but non-culturable cells. As a result, there is a desire for more advanced, cost-effective, and efficient strategies for precisely assessing biofilm presence and characteristics. Artificial intelligence, especially machine learning and deep learning algorithms, has emerged as an effective tool in biofilm research. AI aids in the investigation of biofilm formation, identification of microbial strains inside biofilms, evaluation of environmental impacts on biofilm growth, and the creation of novel anti-biofilm techniques. Compared to previous techniques, AI-driven technologies greatly improve biofilm analysis’s speed, accuracy, and scalability. Future research should combine AI and advanced imaging techniques to understand biofilm dynamics better, enhance detection methods, and speed up the creation of new drugs. Furthermore, investigating the factors that influence biofilm development might lead to innovative preventative techniques that can be used in various contexts, including healthcare and industry.

## Figures and Tables

**Table 1:** Analysis of microbial biofilm on the surface of biotic and abiotic using multiple artificial intelligence-based models.

Name of Pathogens	Surfaces	Name of AI Model	Application of Models in Biofilm Detection	References
*Desulfovibrio alaskensis* G20	Mild steel	M-RCNN	227 times faster than conventional methods	[72]
*Desulfovibrio alaskensis* G20	Mild steel	M-RCNN	1.06 times faster then ImageJ	[111]
*Desulfovibrio alaskensis* G20	Steel	CNN-YOLACT	2.1 times faster then M-RCNN	[113]
*Pseudomonas aeruginosa*, *Staphylococcus aureus*, and *Candida albicans*	Olea europaea leaves	ANN	NA	[117]
*Escherichia coli* and *Salmonella typhimurium*	NA	k-NN, LDA	k-NN better accuracy 90%	[125]
*S. aureus*	Polystyrene plate	RF, LR, SVM, GB, DT, k-NN	GB performed best	[126]
*P. aeruginosa*	Polystyrene plate	RF, LR, SVM, GB, DT, k-NN	GB performed best	[125]
*Bacillus subtilis*	MSgg agar	k-NN, GNB, LR, RF	k-NN performed best	[127]
*S. aureus*, *Acinetobacter baumannii*, *P. aeruginosa*, *Stenotrophomonas maltophilia* and *E. coli*	NA	PCA	95% ACCURACY	[106]
*P. aeruginosa*	NA	oLGBMC	76.92% ACCURACY	[131]
*P. aeruginosa*	Metallic (cast iron) and non-metallic (PVC)	ANN, CNN	high correlation coefficients (0.98 and 0.91) in predicting biofilm thickness for cast iron and PVC	[109]
*Haemophilus influenzae*, *Streptococcus pneumoniae*, *Moraxella catarrhalis*, *P. aeruginosa*, and *S. aureus*	Poly-D-lysine coated chamber slide/glass-bottom dishes (in vitro) and middle ear mucosal surface (in vivo)	VM-RBF, RF, and XGBoost	VM-RBF classifier achieved more than 92% sensitivity, XGBoost shows 90% and 97% sensitivities for the in vitro and in vivo datasets	[27]

## Data Availability

No new data were created or analyzed in this study.
